# Autism across the lifespan: adult care remains inadequate

**DOI:** 10.1016/j.eclinm.2026.103927

**Published:** 2026-04-30

**Authors:** eClinicalMedicine

Autism spectrum disorder (ASD) affects approximately one in 31 children, with prevalence increasing more than threefold over the past two decades. This rise has drawn considerable public attention. However, the equally notable surge in adult diagnoses remains underdiscussed and, although approximately 70% of autistic individuals are adults, services remain disproportionately focused on children. As awareness and acceptance of autism have grown—reflected in annual observances such as Autism Acceptance Month each April—discussion has increasingly shifted toward how acceptance can be translated into long-term accountability for this condition. In line with this, and because core autistic characteristics persist across the lifespan, health systems must adapt to better cater to the needs of adults with the condition and ensure continuity of care from childhood to adulthood.

Several factors contribute to the current situation. First, the significant increase in ASD diagnosis seen since 2000 is now resulting in larger numbers of young people with ASD transitioning into adulthood. Adults who were diagnosed with autism in childhood often experience an abrupt end to support services when they enter adulthood, resulting in a so-called care cliff in health-care access and social support. Structurally, most autism-related programmes are school-based, leading to a loss of services after graduation. Fragmented adult care systems and limited access to providers with appropriate training further exacerbate these challenges. At the same time, the rapidly growing number of adults seeking diagnosis and support is outpacing system capacity, while limited insurance coverage within some health systems compounds health-care access difficulties. In addition, race, gender identity, socioeconomic status, and geography continue to shape who receives an adult assessment and diagnosis and who remains invisible to health systems. These issues exist alongside persistent challenges, such as misdiagnosis (particularly among women), elevated risks of mental ill-health, and increased premature mortality.

One way that health systems need to adapt to caring for adults with autism is by providing autism-friendly communication and clinical care continuously throughout their lifetime, including appointment structures, billing, and insurance models. Standardised training programmes, which have shown some success, are one option for increasing physician knowledge and self-efficacy. Strengthening navigation of the service system to better meet the needs of adults might help to prevent avoidable mental-health crises and inpatient admissions. Diagnostic frameworks must also evolve to better capture the full autistic spectrum, including individuals missed in childhood, female patients, older adults, and individuals without intellectual disability as these groups are often underdiagnosed. Many adult services lack neurodiversity-informed design, contributing to sensory distress, miscommunication, and disengagement from care. Notably, most available evidence comes from high-income countries, representing an evidence gap for lower resource settings.

Current health-care and social support systems often do not meet the growing needs of autistic adults. Adult patients face substantial challenges related to co-occurring mental health problems, social engagement, post-secondary education or employment, daily living skills, and other concerns, but available interventions for improving mental health, social cognition, and overall wellbeing for adults with autism only produce small to moderate effects. Adults with autism frequently report stigma, dismissal and discrimination in health-care settings, alongside an absence of meaningful post-diagnostic support. Young people with ASD can find it difficult to secure a paid job and employment environments often do not foster social integration and workplace wellbeing, and families bear the burden of compensating for systemic failures. These structural gaps ultimately undermine independence, social engagement, and wellbeing across adulthood.

To address these gaps in the support of adults with autism, health and social systems must move toward person-centered, neurodiversity-affirming models of care that ensure continuity across the lifespan and are developed in genuine partnership with people with autism. Priorities include reducing barriers to care through clinician training in autism, creating a more accessible, easy-to-navigate health-care environment that is communicative, supportive, and free from judgment and stigma. Patient Navigators, as implemented in the USA, are another way of facilitating autism-competent care. Investigation of social isolation and discrimination as determinants of mental health, development of community-based, mental-health interventions designed specifically for people with autism, and longitudinal studies identifying modifiable risk factors across adulthood are also needed. Employment research must expand beyond focusing solely on job task training to examine ecosystem-level supports, including workplace accommodations, employer education, and adaptive skills training for adults with ASD. Gaps in daily-living assistance, and post-diagnostic services also demand attention, particularly for under-represented groups. Crucially, future research and policy must embed adults with autism as co-researchers and decision makers, prioritise outcomes that matter to people with autism themselves—such as quality of life, autonomy, and social wellbeing—and develop interventions that are economically viable and culturally adaptable across diverse global contexts.

Adults with autism worldwide face substantial and persistent barriers to diagnosis and to the continuity of support beyond childhood, undermining health, social participation, and independence. Although the causes of this gap are increasingly described, closing it will require coordinated system-level reform, grounded in robust evidence, inclusive research practices, and sustained policy commitment.

